# Study of head trauma patients referred to Shahid Motahhari hospital in Urmia 

**Published:** 2019-08

**Authors:** Nader Aghakhani, Mohammad Delirrad

**Affiliations:** ^*a*^Patient Safety Research Center, Urmia University of Medical Sciences, Urmia, Iran.

## Abstract

**Background:**

Penetrating trauma to the skull is an important cause of death in industrial societies in spite of ‎modern treatments. Most of them have mild and the rest are in moderate or severe trauma. It ‎causes economic, physical and psychological complications for the client and his/her ‎family.100% of severe and 2/3 of moderate cases are led to handicap situations that never ‎treated. The common causes of this problem are car accidents, falling and physical injuries with ‎signs of unconsciousness, drowsiness, epilepsy, nausea, vomiting and headaches. The present ‎study was done on 1796 cases of head trauma in Motahari hospital of Urmia to suggest some ‎guidelines for decreasing of its complication and prevention.‎

**Methods:**

1796 patients who had head injury, were hospitalized in neurology surgical ward of Motahari ‎hospital of Urmia were studied. ‎

**Results:**

Head trauma was the most common cause of hospitalization in neurosurgery ward (40.1%). 162 ‎of samples were female (22.5%) and the rest were male. 35 of them (4, 9%) were dead and 21 of ‎them were from Urmia city. (17%) of dead persons were female. Most of injured persons were ‎from Urmia (53.3%), Khoy (4.6%) and Salmas (4. 2%).Mean age of injurers was 28.53 years ‎and in dead persons was 31.9 years. Most of injuries were in 20-29 years old group. (22. ‎‎6%).Most of injuries was in Farvardin because of increasing car accidents in this month ‎‎(12.8%).‎

**Conclusions:**

The main outcome measure was rates of attendance for head injury. Head injury ratio in men to ‎women was 3.4:1. It may be related to dangerous working environment, driving, and dangerous ‎works for men. The most injured persons were Urmia residents because of population of city, ‎treatment or death of persons in their cities that are not registered in list. The planning and ‎delivery of preventative and management services may be improved by such analyses. Further ‎metacentric work is indicated to map a more accurate clinical picture of head injury survival. ‎Good and rapid road services are useful too.‎

**Keywords:**

Head injury, Urmia, Prevention, Treatment

